# Hip Hop Dance Experience Linked to Sociocognitive Ability

**DOI:** 10.1371/journal.pone.0169947

**Published:** 2017-02-01

**Authors:** Justin W. Bonny, Jenna C. Lindberg, Marc C. Pacampara

**Affiliations:** 1 foundry10, Seattle, Washington, United States of America; 2 Department of Psychology, Morgan State University, Baltimore, Maryland, United States of America; University of British Columbia, CANADA

## Abstract

Expertise within gaming (e.g., chess, video games) and kinesthetic (e.g., sports, classical dance) activities has been found to be linked with specific cognitive skills. Some of these skills, working memory, mental rotation, problem solving, are linked to higher performance in science, technology, math, and engineering (STEM) disciplines. In the present study, we examined whether experience in a different activity, hip hop dance, is also linked to cognitive abilities connected with STEM skills as well as social cognition ability. Dancers who varied in hip hop and other dance style experience were presented with a set of computerized tasks that assessed working memory capacity, mental rotation speed, problem solving efficiency, and theory of mind. We found that, when controlling for demographic factors and other dance style experience, those with greater hip hop dance experience were faster at mentally rotating images of hands at greater angle disparities and there was a trend for greater accuracy at identifying positive emotions displayed by cropped images of human faces. We suggest that hip hop dance, similar to other more technical activities such as video gameplay, tap some specific cognitive abilities that underlie STEM skills. Furthermore, we suggest that hip hop dance experience can be used to reach populations who may not otherwise be interested in other kinesthetic or gaming activities and potentially enhance select sociocognitive skills.

## Introduction

Accumulating evidence suggests that expertise in non-academic areas is connected to cognitive processes. For example, experienced athletes are faster at mentally rotating objects [1] and more highly-skilled poker players have higher performance on working memory tasks [2]. Recent research indicating that video game players outperform novices on tests of visuospatial attention provides evidence that these links are observed with a broad range of informal activities [3]. A smaller body of research that has focused on arts- and humanities-related activities suggest that experience in these domains may also be connected to cognitive as well as sociocognitive processes. For example, individuals with acting experience have been found to outperform novices when inferring the emotion states of others [4]. In light of this evidence, it is important to investigate what types of arts and humanities experiences are linked to cognitive and social skills especially as their availability to students at K-12 schools is at risk of being reduced [5]. In the present study we focused on a particular activity, hip hop dance. This particular dance style has an associated culture and developmental pathway that is relatively unique compared to other types of kinesthetic disciplines. It is also accessible to a wide range of students through the internet and television, and has broad appeal to youth populations [6]. As such, hip hop dance offers an opportunity to investigate whether experience in a relatively popular activity may be related to cognitive and social skills. To address this question we examined whether individual differences in hip hop dance experience were linked to performance on cognitive and social cognition tasks.

### Links between Cognitive Skills and Expertise

There is overlap in the types of cognitive skills that have been found to be connected to individual differences in non-academic activity experience and academic performance. With regard to accumulated experience, previous research provides ample evidence that individual differences in performance on various cognitive tasks are connected to expertise in gaming (e.g., chess and video game players; [7–10]) and fine-motor, or kinesthetic, activities (e.g., athletes, musicians, and dancers; [11]). However, the specific types of cognitive processes linked to accumulated experience varies across areas of expertise. This is believed to be due to specific skills being more heavily recruited by certain activities compared to others. For example, visuospatial skills (e.g., attending to a set of visual targets) have been argued to be especially important when playing video games, such as first-person shooters [3,12,13]. Alongside expertise research, studies have also investigated how individual differences in performance on cognitive tasks are linked with academic outcomes and measures of general intelligence [14–16]. Identifying connections between informal experience, sociocognitive skills, and academic achievement can reveal non-traditional ways to enhance student performance. For example, there is evidence that greater video game experience is connected to higher math achievement scores [17] and adolescents that reported greater levels of video gameplay also reported more positive social experiences [18]. This raises the question of whether other types of informal activities are connected with academic achievement. Across gaming and kinesthetic areas of expertise, three cognitive skills have been repeatedly examined with regard to experience and academic performance: working memory, mental rotation, and problem solving.

#### Working memory

The ability to actively maintain and manipulate information [19,20], working memory in expertise research has typically been assessed using operation span tasks to measure the amount of information one can maintain over a brief period of time (i.e. working memory capacity). Previous research focusing on video game performance has observed that expert video game players tend to score higher on spatial working memory tasks ([21], but see [22]). Relations to working memory performance have also been observed with those that have greater experience in chess [23] and poker [2]. With regard kinesthetic activities, no significant connection has been observed with regard to sports experience and working memory performance [24,25]. Research focusing academic performance has found that students who scored higher on working memory tasks also tended to have higher scores on math achievement tests [16] and that performance on these tasks has also been found to relate to general intelligence [14]. This evidence suggests that accumulated experience in informal activities that draw upon working memory may be connected to individual differences in academic achievement.

#### Mental rotation

Mental rotation ability is typically assessed as the rate at which individuals can determine whether two objects positioned at different angular disparities are the same object or mirror images [26–28]. Expertise research has found evidence that individuals that had greater experience playing certain types of video games responded faster on mental rotation tasks (e.g., first-person shooters, puzzle; [13,29,30]). With regard to kinesthetic experience there is evidence that the link with mental rotation ability varies depending on the type of stimuli being viewed. For mental rotation of artificial objects (e.g., block pieces), similar to results with video game players, students who reported engaging in more sports-related activities had higher performance [31]. However, mixed results have been observed regarding mental rotation of artificial stimuli and accumulated experience in kinesthetic experience ranging from evidence of better performance with greater athletic experience [1], worse performance with greater traditional dance experience (e.g., classical ballet; [32]), to no link with accumulated athletic experience [33]. In contrast, kinesthetic experience has repeatedly been found to correlate with performance when mentally rotating natural stimuli, such as animals [34], but more commonly human figures and body parts [33,35]. This suggests that the effects of kinesthetic expertise may only extend to embodied stimuli, where the individual can spatially map their body to a rotated object [33,35]. Individual differences in mental rotation ability have also been found to be connected to academic performance, in particular math ability. Specifically, students who have higher performance on tasks that tap spatial abilities, including mental rotation, score higher on math achievement tests [36], suggesting that spatial abilities are relied upon when performing specific academic activities [15].

#### Problem solving

Although it can be defined multiple ways, problem solving skill is typically assessed as the ability of individuals to identify a strategy, and plan the steps required, for transforming an initial state to a goal state [37]. Although less research has focused on problem solving ability, compared to working memory and mental rotation, with regard to expertise, positive results have been observed with gaming experience. Specifically, individuals with more experience in chess are more likely to find the optimal solution to a problem [38] and individuals who have more video game experience rate their problem solving abilities higher than those who have less experience [39]. Connections have also been observed between problem solving ability and academic achievement. Specifically, individuals who displayed more efficient problem solving performance (e.g., require fewer steps to get from start to goal state) also scored higher on tests of reading ability [40] and math ability [41]. This evidence suggests that both gaming expertise and academic achievement are connected to problem solving ability. However, it remains to be determined whether individuals who vary in accumulated kinesthetic experience also perform differently on tests of problem solving ability.

### Links between Social Cognition Skills and Expertise

In addition to cognitive skills, there is evidence that social processing ability is connected to both informal experience and academic success. However, unlike the cognitive abilities discussed above, the majority of this research has focused on kinesthetic experience, more specifically dance expertise. When examining the impact of accumulated dance experience, research has typically focused on the sociocognitive ability to simulate and represent the actions and mental states of others. For example, professional classical ballet and capoeira dancers had stronger activation in brain regions associated with planning and executing motor actions when viewing a dance routine in the dance in which they specialized [42]. Similarly, when viewing point-light displays of dancers, participants with experience in classical ballet had greater recognition performance when the display contained ballet-specific movements [43]. This neural and behavioral evidence is in line with the view that motor actions are interpreted using one’s own movement repertoire [44]. In addition to dance, connections between action recognition task performance and kinesthetic experience have been observed in other domains such as basketball [45] and rugby [46]. This suggests that in addition to links with cognitive performance, accumulated experience in kinesthetic activities is related to sociocognitive performance.

Although these studies suggest that kinesthetic experts are better able to recognize the actions of others, these effects were specific to representing the mental states of others within their area of expertise. Additional research provides indirect evidence that these connections may extend to other sociocognitive processes with stimuli that are not specific to an area of expertise. Although still specific to a particular field, there is evidence that expert basketball players are better able to detect deception in other players, such as judging whether an opponent is feinting a basketball move [47]. Partial evidence of mental state recognition extending outside an expert’s specific field comes from arts and humanities, specifically acting. Experienced actors have been found to be more accurate at recognizing the emotion states of others when completing the ‘Reading the Mind in the Eyes’ (RME) task [4]. For this task, individuals are shown images of human faces displaying emotions that are cropped to only reveal the immediate area around the eyes and have to pick which of four emotion words is being displayed. Previous research suggests that performance on this task requires engaging in emotion processing and theory of mind [48]. When high school students were given training in either acting or visual arts, students in the acting condition displayed marginal gains in theory of mind performance, as well as empathy ability, compared to students enrolled in visual arts training [49]. When a similar training program was presented to elementary school students, gains in empathy were only observed for those in the acting condition [49]. Given the connections observed between the ability to recognize the mental states of others and motor and acting expertise, an open question is whether individuals experienced in kinesthetic activities would display advantages in mental state recognition. In addition to kinesthetic experience, sociocognitive skills have also been found to correlate with academic achievement. Specifically, studies have examined whether students’ ability to recognize and represent the mental states of others is connected to reading achievement. It has been found that young students who had better performance on a false-belief task also scored higher on standardized tests of reading comprehension and language ability, suggesting that recognizing the mental states of others contributes to academic achievement [50,51].

### Hip Hop Dance Experience and Sociocognitive Skills

An area of expertise that has received relatively little attention with regard to individual differences in sociocognitive skills and academic achievement is hip hop dance. As a dance style, hip hop is growing in popularity with an increasing number of television shows and movies [52]. That hip hop dance is popular among youth from underrepresented minorities and lower socioeconomic statuses make this type of informal activity a potential way to reach this population. Indeed, hip hop dance has been used in a number of interventions designed to address obesity and health issues in minority children [6,53] and enhance self-esteem of minority children in school [54]. Despite its prevalence and use with under-represented groups, there has yet to be an investigation, to our knowledge, into to whether hip hop dance experience is connected to individual differences in cognitive and social skills. This is especially important when considering that individuals from lower socioeconomic statuses are at risk for failing to meet education standards [55]. Furthermore, there is evidence that cognitive skills, such as problem solving ability, mediate the relation between student achievement and socioeconomic status [56]. Examining the extent to which hip hop dance experience is connected to sociocognitive skills can inform whether future research incorporating hip hop dance interventions could lead to improvements in these specific areas.

The cultural variability and informal developmental pathways make hip hop dance somewhat unique compared to other more traditional dance styles. Hip hop dance initially developed in African American communities and incorporates a number of structured and unstructured actions and movements [57]. It is more accessible, compared to other classical dance styles, to a wider population due to its typically informal developmental pathway. For example, many hip hop dancers typically begin learning skills via self-teaching or practicing with friends at home as well as with community groups and clubs (personal communication). In contrast, to learn a traditional dance style such as ballet or jazz, dancers typically enroll in workshops and classes at dance studios. Compared to other dance genres, such as classical ballet, the style of hip hop dance developed relatively recently and is constantly evolving, with many geographic variations in the types of actions and moves utilized [52,58]. The culture surrounding hip hop is also significantly different compared to traditional dance styles, with different styles of music, racial, and socioeconomic groups [58]. As such, it is unclear whether connections between sociocognitive skills previously observed with other kinesthetic areas of expertise would be observed with hip hop dance experience.

To determine whether hip hop dance experience may be connected to mental processes underlying academic performance, we examined whether accumulated experience in this activity was linked with individual differences in working memory, mental rotation, problem solving, and social cognition skills. In line with previous research, these skills may be connected to hip hop dance experience for the following reasons. With regard to working memory, when learning or developing choreography for a routine, hip hop dancers have to not only coordinate multisensory information (e.g., music, kinesthetic, visual) but also have to maintain their spatial position on a dance floor according to a predetermined sequence of movements. Similar to video game players, we predict that hip hop dancers with greater experience will score higher on spatial working memory tasks. Hip hop dancers, along with other styles, will typically learn a routine by mirroring the movements of a choreographer. As such we predict that dancers with greater hip hop experience will have better performance on mental rotation tasks. We also predict that problem solving skills, specifically planning, will be linked to dance experience given that choreographing a dance routine requires planning the movements and timing as it relates to the tempo of music. Similar to other arts-related activities, we predict that hip hop dance experience will be connected to sociocognitive skills, specifically theory of mind ability. Hip hop performers many times need to identify emotion states that they want to convey to others through their dance routine as well as the emotions that are to be displayed by other dancers (personal communication). We predict that experience engaging in these mentalizing activities will be reflected in better recognition of mental, specifically emotion, states of others.

An open question is whether hip hop dance experience is differentially linked to sociocognitive skills compared to experience in traditional dance areas. Although there are differences, as discussed above, overall, dancers are engaging in a kinesthetic activity. Indeed, there are many individuals who practice in multiple dance styles, including hip hop and classical styles (personal communication). Since previous research has focused primarily on classical dance styles, the present study can inform the extent to which there is overlap in some sociocognitive skills amongst these dance styles. Furthermore, the present study can evaluate the extent to which hip hop dance and other dance styles are linked to specific sociocognitive processes.

#### Present study

In the present study we examined whether hip hop dance experience was connected to cognitive and social skills. We focused specifically on skills that have been previously investigated in gaming and kinesthetic expertise research and may be relied upon during hip hop dance: working memory, mental rotation, problem solving and recognizing emotional states of others. To do so, we recruited dancers who varied in levels of experience across different dance styles. We defined dance experience as the number of estimated hours that individuals had spent either deliberately practicing or teaching choreography to other dancers. We included both types of experience due to evidence that while deliberate practice is a major determinant of expertise, it is not the only significant factor [59–61]. To assess sociocognitive skills, we utilized computerized decision tasks, specifically the symmetry operation span task [62,63], block and hand mental rotation tasks [27,28], the Tower of London task to assess problem solving ability [37], and the Reading the Mind in the Eyes (RME) task to assess emotion recognition skill [48]. To examine if hip hop dance specifically, and dance experience more generally, was linked with performance on the tasks, we examined if dance experience predicted performance and interacted with task-specific factors that have previously been found to impact performance. We also controlled for the age and gender of participants since performance on a number of the included measures have been found to differ between males and females [31,48]. We used linear mixed models to analyze performance as these types of models are able to account for variation in performance across participants and stimuli and are relatively robust to unbalanced data [64–67]. We hypothesized that hip hop experience would be positively correlated with working memory capacity, rate of optimal problem solving, and accuracy in recognizing the emotion states of others. We also hypothesized that hip hop experience would be correlated with faster response times on mental rotation judgments and that this may differ when making judgments about block figures versus body parts.

## Method

### Participants

A total of 61 participants (45 females, 16 males) were included in data analysis. Participants were from a wide age range (*M* = 23.8 years, *SD* = 6.9, *Min* = 12, *Max* = 55) and different racial and ethnic backgrounds (*N* = 59 provided racial and ethnic information; Asian = 22; African American = 1; Pacific Islander = 1; Pacific Islander + Hispanic = 2; White = 23; White + Hispanic = 1; Multiracial = 9).

Participants were recruited from three locations within Seattle, Washington, United States of America that were identified as likely to be frequented by experienced dancers. One location was a local dance studio that offered a range of dance classes to individuals of different ages. A second location was the dance department at a regional fine arts college and the third location was a student hip hop dance club at a large public university. Both adults and minors were recruited for the present study and provided written consent, and assent if required. For participants less than 18 years of age, written consent from the parent or guardian and written assent from the participant was required. The protocol for the present study was approved by a local institutional review board composed of representatives from the professional and surrounding communities (foundry10; protocol number 2015.001) and abided by the Declaration of Helsinki. Participants were provided with a $10 gift card for their participation in the study.

### Hardware and Software

All components of the study were presented via a set of tasks built using the Python-based experiment builder OpenSesame (v. 2.9.6; [68]) and custom scripts. Laptops running the Windows 8 operating system were used to conduct the study (screen diagonal of 13.3 in). A total of five laptops were used to run up to five participants simultaneously on-site at each of the locations. Laptops were arranged on a table setup in a space adjacent to dance sessions allowing participants to complete the study when they were not dancing.

### Dance Experience Questionnaire

A common method for measuring experience within a specific domain is to have individuals retrospectively estimate the number of hours they spent practicing the activity of interest. This is typically done using a questionnaire where participants estimate the youngest age at which they started practicing an activity and then have them estimate the number of hours per week that they spent practicing that activity over two or three year periods [69,70]. Taking a similar approach, we asked participants to estimate the youngest age at which they began to deliberately practice hip hop dance or other dance styles (e.g., dancing to learn or refine a skill or routine) as well as the youngest age at which they began teaching hip hop dance or other dance styles (e.g., teaching a dance class or training dancers for a performance). We limited the minimum age at which they could indicate they taught a dance style to 11 years based on conversations with experienced dancers which indicated it was highly unlikely that teaching younger than that age was at the same level as instruction performed by older individuals (this was confirmed by dancers reporting the youngest age they began teaching was 12 years of age). An additional set of Likert-type questions asked participants to rate their perceived expertise in hip hop and other styles of dance on a 0 to 8 scale (e.g., “Indicate to what extent you are a novice [0] or professional [8] hip hop dancer”). The questionnaire also assessed demographic information including age, race, ethnicity, and gender.

We defined dance experience as the total number of hours an individual spent deliberately practicing and teaching dance. As such, we combined estimated number of hours deliberately practicing hip hop and teaching hip hop into total hip hop dance hours and likewise for other dance styles.

### Spatial Working Memory Task

The selected working memory task has been frequently used in previous research to assess the ability of individuals to retain visuospatial information over a brief period of time while performing a diversion task. It was based on the abbreviated and automated versions of the symmetry operation span task which sequentially presents participants with a series of blocks placed within a spatial grid and asks them to recall the position and order of each block [62,71]. To maximize the chance that performance on the task is based on visuospatial working memory, between each presentation of the block within a grid, participants had to judge whether a black-and-white figure was symmetrical along the vertical axis. This diversion judgment was included to reduce the chance that participants could use rehearsal mechanisms to retain visuospatial information.

**Fig 1:**
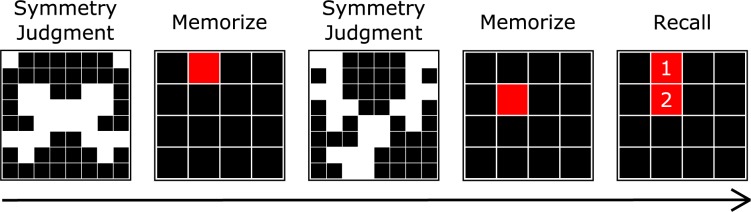
Trial procedure for spatial working memory task. Participants were asked to remember and then recall the order and locations of red blocks. Symmetry judgements were made between each block presentation.

The trial procedure was as follows: a fixation dot appeared for 800 ms after which an 8 x 8 black-and-white figure was presented. Participants had to judge whether the black-and-white figure was symmetrical along the y-axis (approximately half of the figures were symmetrical) within a fixed response period (see Fig 1). The duration of the response period depended on each participant's performance during the previously administered practice trials. Following previous research, participants were presented with six practice trials performing the symmetry task where the symmetrical figure remained onscreen until they clicked one of two buttons to indicate whether it was or was not symmetrical [72]. The mean reaction time was calculated for these trials and the duration for symmetry judgments on test trials was set as the mean plus 2.5*SD. Participants were informed of the time in which they were required to respond prior to the start of test trials. After the symmetry judgment, a 4 x 4 grid with one randomly placed red block was presented for 650 ms. Immediately after this presentation, either the next symmetry judgment was displayed, if the number of pairs for the trial had yet to be reached, or, the response period was initiated. The symmetry judgment and red block pairs occurred from 3 to 5 times (a total of 2 complete trials for 3-, 4-, and 5-item sets; 24 individual block responses total). At the end of a set, participants were presented with a blank 4 x 4 grid and were instructed to click the boxes that contained the red blocks in the exact order in which they were presented using a computer mouse. There was no time limit for the response period and immediately after the last response the next trial began.

Previous research has found that participants could complete the entire task in around three to five minutes and that performance on the task is valid and reliable (correlation with latent working memory capacity variable = 0.48; Cronbach's alpha = 0.59; [71]). Scoring for the working memory task followed the absolute scoring method which summed the number of blocks correctly recalled in position and order [20]. Accuracy on the symmetry task was used as a method for determining whether participants were following directions for each trial which, following previous research, was indicated by accuracy less than 85% [62]. The dependent variable generated for this task was block score, the total number of blocks correctly recalled for a trial.

### Mental Rotation Task

**Fig 2:**
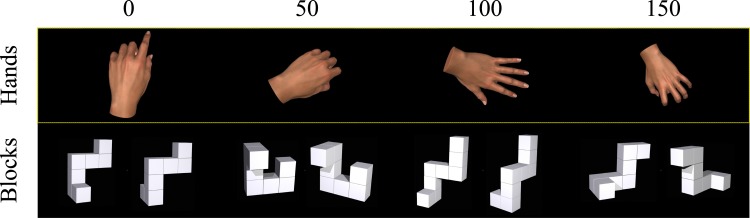
Example stimuli used for the mental rotation task. Participants were asked to compare the orientation of a stimulus image to either a second image (block condition) or vertical orientation (hand condition). Orientations varied across trials (0°, 50°, 100°, 150°). Stimulus images for the block condition were from Ganis and Kievit (2015).

The goal of this task was to assess the ability of participants to mentally rotate objects and determine if performance differed according to whether the objects were artificial, specifically a block figure, or a body part, specifically a hand. For the task, participants were presented with two sets of judgments for images of block figures and hands. The block condition was modeled after Ganis and Kievit (2015) and had participants judge whether side-by-side Tetris-like blocks rotated by a specific angle were the same or mirror images of each other (see Fig 2). For the hand condition, participants were presented with either a left or right hand at a specific orientation and had to judge whether the hand was indeed left or right (see Fig 2). Stimuli sets for the block and hand conditions were computer generated 3D images: block images were from stimuli sets made available by Ganis and Kievit (2015), and hand images were generated using multiple hand positions via a virtual 3D model. For both conditions the stimuli were rotated by one of four orientations (0°, 50°, 100°, 150°) with rotation defined for the block condition as the orientation of the left block compared to the right block and for the hand condition defined as the angle offset from vertical orientation (i.e. fingers pointing vertically; see Fig 2).

The procedure for the task was as follows. Similar to previous research, on each trial participants were first presented with a fixation point (350 ms) after which the mental rotation stimulus appeared [27]. The stimulus remained on screen until participants made a key press regarding the judgment. For the block condition, participants were asked to judge whether the two block images were of the same, but rotated, object (‘S’ key) or different objects that were mirror images of each other (‘D’ key). For the hand condition, participants judged whether the object presented was a left (‘Q’ key) or right (‘P’ key) hand. When considering the hand stimuli, participants were asked to imagine that the hands represented their own. The stimulus remained onscreen until participants made a response or after 7.5 seconds had expired, after which the next trial began. Each condition was presented in separate blocks of trials (order randomly determined across participants) and prior to the start of each block of trials participants completed four practice trials. Within each block, a total of six trials were presented for each orientation (24 trials total for each stimulus condition), the order of which was randomly determined. The dependent variable generated for this task was reaction time (RT) in ms for making button-press responses. We also recorded the accuracy of each judgment to be used in analyses.

### Tower of London Task

**Fig 3:**
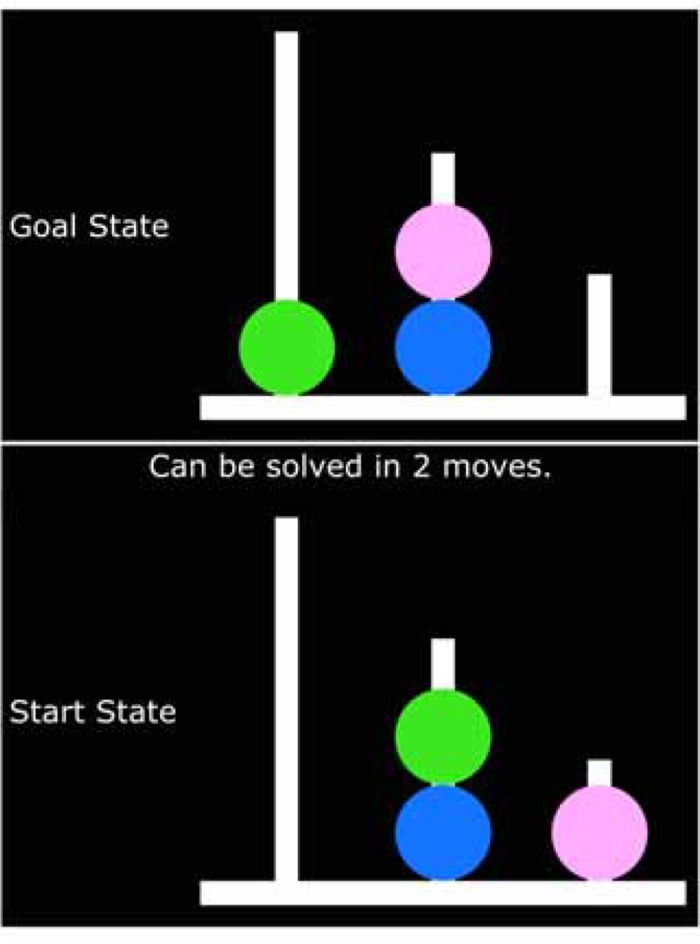
Example trial from the Tower of London task. Participants had to plan and execute the steps required to get the marble positions from the start state to those of the goal state.

The Tower of London task has been used in previous research to assess problem solving and planning abilities [37,73]. The setup of the task was as follows. In a virtual space three pegs of different heights were arranged on a board with three different color marbles of the same size placed on the pegs in a specific arrangement. The heights of the pegs correspond to the number of marbles that could fit; the tallest could have three marbles, the shortest could have one marble and the middle-height peg could have two marbles. The participant’s task was to move the marbles across the three pegs to match a displayed goal board (see Fig 3). Participants were asked to plan the sequence of moves required before making their first move and to make as few moves as possible to match the goal board.

For each trial, participants were first presented with a blank (only pegs, no marbles) “start” board, the board which they will manipulate, and a blank “goal” board positioned above and were asked to use a mouse to click an overlaid “ready” button to begin the trial. Once clicked, the complete start and goal boards were displayed and a text display informed participants of the minimum number of moves required to get the start board to match the goal board. Participants then used the mouse to move the marbles across pegs in order to make the start board match the goal boards; a left-click on a marble allowed the participant to move it (attached to mouse cursor) and a subsequent left-click on a peg that was available (e.g., had enough space for the marble) placed the marble on that peg (the task prevented the participant from making moves that were against task rules). Once the start board matched the goal board or 60 seconds had elapsed, participants were provided feedback for 1500 ms regarding either how many moves it had taken them to match the boards or that they ran out of time. Participants completed two practice trials (one trial that could be solved in two moves, another that could be solved in four moves) prior to being presented with six test trials, two trials for each minimum move lengths of four, five, and seven (presented in random order; see S1 File for specific start and goal board arrangements). Following previous research, the dependent variables generated for this task were the number of extra moves, with regard to the minimum number of moves to complete a trial; the duration between initial presentation of start and goal states and first placement of a marble on a peg was also recorded to be included as a predictor in analyses [37]. Following previous research, only complete trials were included in data analysis; trials for which participants ran out of time were discarded.

### Reading the Mind in the Eyes Task

The Reading the Mind in the Eyes (RME) task was used to assess the degree to which participants could recognize the emotion states of others [48]. Participants were asked to select which of four emotion words best matched the emotion displayed by a picture of a human face cropped such that only the eyes and space between and immediately around the eyes were visible. For each trial participants were presented with an image displaying a particular emotion centered on a computer screen and four emotion words adjacent to the image (top-left, top-right, bottom-left, bottom-right; e.g., a cropped image of a “panicked” face surrounded by the words “jealous”, “arrogant”, “hateful”, and “panicked”). Participants were asked to use the mouse cursor to click which word best matched the emotion displayed by the image as quickly and as accurately as possible and once a response was made the next stimulus was immediately presented. There were a total of 36 test trials presented in a fixed order and one practice trial. Previous research has found differences in performance on the RME according to whether the target emotion was positive, neutral, or negative in valence [74,75]. Specifically, it has been found that performance for negative and positive emotion images differ from each other as well as from neutral emotion images [74,75]. As such we used the coding scheme developed by Harkness and colleagues (2005) to categorize the valence of trials as positive (*N* = 8), neutral (*N* = 16), or negative (*N* = 12), and examined whether performance varied according to emotion condition. The dependent variable generated for this task was accuracy (0 = incorrect emotion word selected; 1 = correct emotion word selected). We also recorded reaction times such that they could be included in analyses.

### Study Procedure

At each of the venues, individuals were invited to participate in the study using informational flyers and verbally asking them before and after dance sessions. Those who expressed interest in participating were provided with informed consent forms approved, along with the research protocol, by an institutional review board. Individuals who were 18 years of age or older provided written consent while those younger provided written assent along with the written consent of their parent or guardian. Participants were then provided the tasks and questionnaire in the following order: working memory task, mental rotation task, Tower of London, RME task, and questionnaires. After completing the set of tasks, which lasted approximately 25 minutes, participants were provided with an incentive and thanked for their participation.

### Statistical Analyses for Task Performance

For each task we analyzed performance using linear mixed models due to their robustness to unbalanced data as well as ability to account for individual differences with regard to performance using random and fixed effects [64,65]. Following the recommendations of Baayen and colleagues (2008) and Barr and colleagues (2013), for each task we ran a maximal linear mixed model for each dependent variable including each valid trial completed by each participant using the maximum likelihood method, using ‘lme4’ version 1.1–11 [76–78] running in the R version 3.2.4 environment [79]. We included participant and task factors as random slopes and intercepts and then determined whether the model was over-specified with regard to random effects [64]. For each task, hip hop dance experience and other dance style experience were included as main effects as well as interaction terms between task-specific variables. Age and gender were controlled for by including each factor as a main effect in the model. For tasks that included multiple indicators of performance (mental rotation: reaction time and accuracy; Tower of London: extra moves and reaction time; RME task: accuracy and reaction time) the secondary performance indicator was included as a predictor to control for speed-accuracy trade-offs. All continuous predictor variables were centered and categorical predictors were dummy coded (gender contrast coded such that females = -1, males = 1).

We used the fitted linear models to evaluate significant effects across two steps. First, to determine whether there were effects or interactions between independent variables we computed a mixed-factor analysis of variance (ANOVA) table populated by the fitted linear mixed model. The ANOVA statistics and degrees of freedom were estimated via linear mixed models using the Satterthwaite approximation method via the ‘*lmerTest’* version 2.0–30 package [80]. If there was a significant effect or interaction, in the second step the detailed linear mixed model was utilized to determine which factor levels were driving the effect (the full linear mixed models are presented in Supporting Information S1 File). Effect sizes for ‘*lmer’* models were estimated using *Ω*^*2*^_*0*_ an approximation of variance explained by a model [81]. For all reaction time-based tasks, trials for which RT was shorter than 100 ms were identified as spurious responses and were discarded [82]. The ‘psych’ version 1.5.8 package was used to generate descriptive statistics for dependent variables [83]. Details regarding the selection of maximal or reduced linear models and the reported effect sizes are provided in Supporting Information (S1 File).

## Results

### Dance Experience Questionnaire

**Fig 4:**
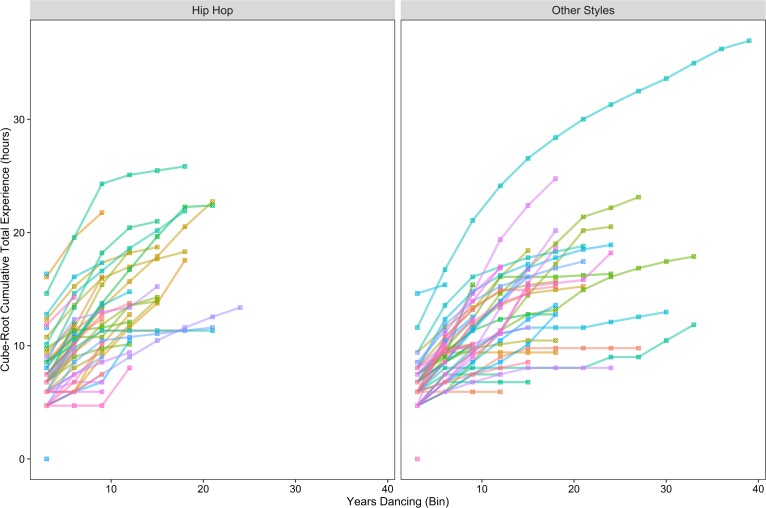
Accumulated dance experience by age. Accumulated hip hop and other dance style experience (cube-root of total accumulated hours) as a function of number of years dancing each type of dance (binned by three-year increments). Each colored line segment indicates the accumulated experience of a specific participant.

One participant failed to answer questions regarding the number of hours spent practicing and teaching hip hop dance on the dance experience questionnaire. These missing values were estimated using a linear regression that included all other participants with start age of dance practice and self-ratings of hip hop dancing and teaching proficiency as dependent variables. Participants varied in the amount of dance experience they had accumulated (see Table 1). As discussed previously, we defined overall dance experience as the combination of total number of hours deliberately practicing dance and total number of hours teaching dance. A slight majority of participants had accumulated more experience with hip hop dance compared to other dance styles (33 out of 61). To account for substantial positive skew, in all analyses cumulative dance experience measures were cube-root transformed (see Fig 4). The total number of hours of dance experience significantly correlated with self-reported ratings of dance experience (hip hop: *r*[59] = 0.783, *p* < 0.001; other styles: *r*[59] = 0.732, *p* < 0.001) providing evidence that total number of hours was a valid measure of dance experience. There was a significant negative correlation between total hip hop dance hours and other style dance hours, *r*(59) = -0.412, *p* = 0.001.

**Table 1:** Descriptive statistics of dance experience measures (no transformations applied) as assessed by the dance experience questionnaire (*N* = 61).

Dance Experience Measure	*M*	*SD*	*Median*	*Min*	*Max*	*Skew*
Hip Hop Practice (hours)	2269.75	2832.54	1456	0	15392	2.38
Other Styles Practice (hours)	2804.97	3387.49	1664	0	18512	2.31
Hip Hop Teaching (hours)	517.55	1117.04	104	0	5616	3.05
Other Styles Teaching (hours)	814.95	4091.89	0	0	31928	7.20
Hip Hop Total (hours)	2787.30	3497.08	1560	0	17264	2.04
Other Styles Total (hours)	3619.92	6848.36	1664	0	50440	5.32
Self Hip Hop Experience Rating	3.66	2.10	4	0	8	-0.17
Self Other Styles Experience Rating	3.16	2.53	3	0	8	0.13

### Working Memory Task

#### Data reduction

Following previous research, trials for which the average accuracy for the symmetry judgments was lower than 85% were discarded since it was likely that participants were engaging in rehearsal strategies to remember block locations [62,71,72]. Next, trials for which the median block RT was greater than or less than 3*SD from the mean were discarded (see Table 2 for descriptive statistics). This left a total of 335 valid trials (31 trials discarded) across participants. The remaining trials were used to evaluate linear mixed models predicting block score with set length (number of block–symmetry judgment pairs) as the task-specific independent variable.

**Table 2:** Descriptive statistics for trial performance for each decision task.

	Working Memory	Mental Rotation	ToL	RME
	*Block Score*	*RT*	*Extra Moves*	*Accuracy*
*All valid trials*	366	2928	332	2196
*N Rejected*	31	114	13	52
*% Retained*	90.746	95.949	95.925	97.575
*Mean*	2.952	2032.461	3.056	0.752
*SD*	1.342	1490.289	3.5	0.432
*Median*	3	1538.000	2	1
*Min*	0	440	0	0
*Max*	5	7419	14	1
*Mean Trials per Participant*	5.583	46.131	5.23	35.148
*Min Trials per Participant*	2	24	2	28
*Max Trials per Participant*	6	48	6	36

RT, response time; ToL, Tower of London; RME, Reading the Mind in the Eyes

#### Analyses

A maximal linear mixed model with age, gender, set length (3-item set as reference), hip hop dance experience, and other dance style experience as fixed effects, random slope and intercept for participant (by set length) was analyzed. Using the model, a mixed-factor ANOVA was calculated and revealed a significant main effect of set length, *F*(2, 133.661) = 3.854, *p* = 0.024 (see Table 3). Using the detailed linear mixed model (see Table A in S1 File), the main effect was driven by a significantly more blocks recalled for the 4-item, *t*(245.342) = 2.702, *p* = 0.022, compared to the 3-item set length trials (Bonferroni-corrected for three post-hoc contrasts; 5-item versus 3-item set length trials corrected *p*-value = 0.101).

**Table 3:** Main effects and interactions on the working memory task when predicting block score. A linear mixed model was used to compute a mixed-factor ANOVA.

Effect	SS	MS	df Num.	df Denom.	*F*-value	*p*-value
Age	0.062	0.062	1	121.39	0.087	0.768
Gender	1.061	1.061	1	114.562	1.502	0.223
Hip Hop Hours	0.167	0.167	1	70.358	0.237	0.628
Set length	5.441	2.721	2	133.661	3.854	0.024*
Other style Hours	1.400	1.400	1	76.232	1.983	0.163
Hip Hop Hours: Set length	0.228	0.114	2	131.704	0.161	0.851
Hip Hop Hours: Other style Hours	0.000	0.000	1	74.253	0.00	0.998
Other style Hours: Set length	0.639	0.320	2	147.656	0.453	0.637
Hip Hop Hours: Other style Hours: Set length	0.232	0.116	2	136.551	0.164	0.849

SS, sum of squares; MS, mean square; df Num., degrees of freedom in numerator; df Denom., degrees of freedom in denominator

*p<0.05

### Mental Rotation Task

#### Data reduction

For each condition (hand, block), trials for which reaction time was greater than or less than 3*SD from the mean were discarded (see Table 2 for descriptive statistics). Additionally, one participant had mean accuracy on the hand condition less than 3*SD the mean and one participant had no correct answers on the block condition; trials for each participant were removed from the respective conditions. The remaining trials were used to evaluate linear mixed models predicting reaction time with orientation and stimulus type as the task-specific independent variables while controlling for the accuracy of judgments.

#### Analyses

**Fig 5:**
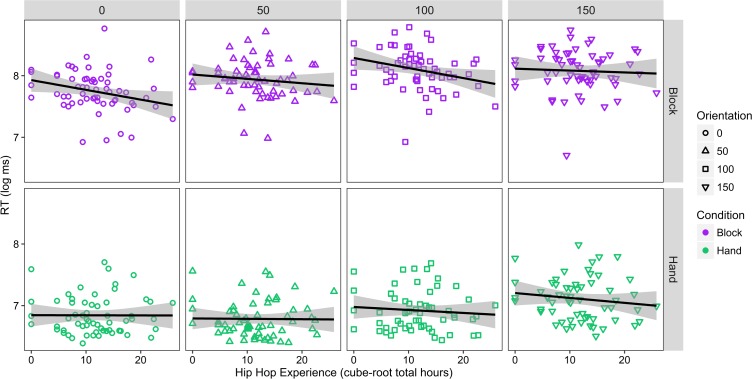
Relation between mental rotation task performance and hip hop dance experience. Scatter plot of mental rotation task reaction time (RT) and total hip hop hours across condition (block, hand) and orientation (0°, 5°, 100°, 150°). Across participants, as hip hop experience increased, RTs decreased for the 150° hand condition relative to the 0° hand condition. Shaded areas indicate the 95% CI for the regression line.

Due to positive skew (skew = 1.34), RTs were log-transformed in subsequent analyses (transformed skew = 0.26). A maximal linear mixed model with age, gender, orientation (reference for orientation = 0°), condition, hip hop dance experience, accuracy, and other dance style experience as fixed effects, random slope and intercept for participant (by orientation, stimulus type, and accuracy) and random intercept for stimulus image was analyzed. Using this model, we computed a mixed-factor ANOVA to examine whether there were main effects and interactions between predictor variables. We observed significant main effects of gender (shorter RTs for males versus females), accuracy (shorter RTs for incorrect trials), orientation, condition (shorter RTs for hand versus block trials), and significant interactions between orientation and condition and between hip hop hours, condition, and orientation, (see Table 4). The detailed linear mixed model (see Tables B and C in S1 File), revealed that the two-way interaction between condition and orientation was driven by shorter RTs for the 100° orientation hand condition, estimate = -0.398, *t*(41.083) = -2.553, *p* = 0.043 (Bonferroni-corrected for six post-hoc contrasts, all other *p*s > 0.091) compared to the reference (block condition, 0° orientation). The three-way interaction was driven by a significant connection between hip hop dance experience and reaction time for the hand condition and 150° orientation, estimate = -0.158, *t*(2623.253) = -2.869, *p* = 0.033 (Bonferroni-corrected for eight post-hoc contrasts). This interaction indicated that individuals with more hip-hop expertise had shorter RTs for the 150° orientation in the hand condition compared to the respective references (block condition, 0° orientation; see Fig 5).

**Table 4:** Main effects and interactions on the mental rotation task when predicting reaction time. A linear mixed model was used to compute a mixed-factor ANOVA.

Effect	SS	MS	df Num.	df Denom.	*F*-value	*p*-value
Age	0.413	0.413	1	62.449	1.897	0.173
Gender	1.695	1.695	1	61.151	7.794	0.007**
Accuracy	1.365	1.365	1	50.797	6.274	0.016*
Orientation	6.886	2.295	3	41.845	10.552	<0.001***
Condition	66.559	66.559	1	91.497	305.98	<0.001***
Hip Hop Hours	0.01	0.01	1	61.736	0.044	0.834
Other style Hours	0.11	0.11	1	61.248	0.508	0.479
Orientation: Condition	1.861	0.62	3	40.329	2.851	0.049*
Orientation: Hip Hop Hours	1.097	0.366	3	268.457	1.681	0.171
Condition: Hip Hop Hours	0.052	0.052	1	60.923	0.238	0.628
Orientation: Other style Hours	0.805	0.268	3	265.701	1.233	0.298
Condition: Other style Hours	0.144	0.144	1	61.833	0.663	0.419
Hip Hop Hours: Other style Hours	0.354	0.354	1	66.871	1.629	0.206
Orientation: Condition: Hip Hop Hours	2.202	0.734	3	2611.166	3.374	0.018*
Orientation: Condition: Other style Hours	0.278	0.093	3	2614.294	0.426	0.735
Orientation: Hip Hop Hours: Other style Hours	0.489	0.163	3	260.886	0.75	0.523
Condition: Hip Hop Hours: Other style Hours	0.447	0.447	1	65.631	2.055	0.156
Orientation: Condition: Hip Hop Hours: Other style Hours	0.837	0.279	3	2606.223	1.283	0.278

SS, sum of squares; MS, mean square; df Num., degrees of freedom in numerator; df Denom., degrees of freedom in denominator

*p<0.05

**p<0.001

***p<0.001

### Tower of London Task

#### Data reduction

Following previous research, trials for which participants ran out of time before providing a solution were discarded [37]. Next, trials for which the first move duration or number of moves made was greater than or less than 3*SD from the mean were discarded (see Table 2 for descriptive statistics). This left a total of 332 valid trials (13 trials discarded) across participants. The remaining trials were used to evaluate linear mixed models predicting number of extra moves using first move duration and minimum number of moves as the task-specific independent variables.

#### Analyses

A reduced linear mixed model with age, gender, minimum number of moves (4 moves used as reference), first move RT, hip hop dance experience, and other dance style experience as fixed effects and random intercepts for participant and stimulus type, was analyzed. Using the model, a mixed-factor ANOVA was calculated and revealed a significant main effect of first RT (fewer extra moves with longer RTs), *F*(1, 265.172) = 3.930, *p* = 0.001, and minimum moves, *F*(2, 8.877) = 19.130, *p* < 0.001 (see Table 5). Using the detailed linear mixed model (see Table D in S1 File), the main effect of minimum moves was driven by significantly more extra moves made for the 5-minimum, *t*(8.678) = 6.007, *p* = 0.001, and 7-minimum, *t*(8.521) = 4.147, *p* = 0.008, trials compared to the 4-minimum trials (Bonferroni-corrected for three post-hoc contrasts).

**Table 5:** Main effects and interactions on the Tower of London task when predicting number of extra moves. A linear mixed model was used to compute a mixed-factor ANOVA.

Effect	SS	MS	df Num.	df Denom.	*F*-value	*p*-value
Age	1.774	1.774	1	68.961	2.214	0.141
Gender	0.449	0.449	1	53.379	0.561	0.457
First RT	3.150	3.150	1	265.172	3.930	0.048*
Hip Hop Hours	0.606	0.606	1	53.992	0.756	0.388
Minimum Moves	30.667	15.334	2	8.877	19.130	0.001***
Other style Hours	0.674	0.674	1	59.584	0.841	0.363
Hip Hop Hours: Minimum Moves	0.079	0.040	2	258.254	0.049	0.952
Hip Hop Hours: Other style Hours	0.480	0.480	1	79.304	0.599	0.441
Other style Hours: Minimum Moves	4.041	2.021	2	262.374	2.521	0.082
Hip Hop Hours: Other style Hours: Minimum Moves	2.621	1.310	2	276.700	1.635	0.197

RT, reaction time; SS, sum of squares; MS, mean square; df Num., degrees of freedom in numerator; df Denom., degrees of freedom in denominator

*p<0.05

***p<0.001

### Reading the Mind in the Eyes Task

#### Data reduction

Of a total of 2196 valid trials across participants 52 trials having reaction times greater than or less than 3*SD from the mean were discarded (see Table 2 for descriptive statistics). The remaining trials were used to evaluate linear mixed models predicting accuracy with target emotion and reaction time as the task-specific independent variables.

#### Analyses

**Fig 6:**
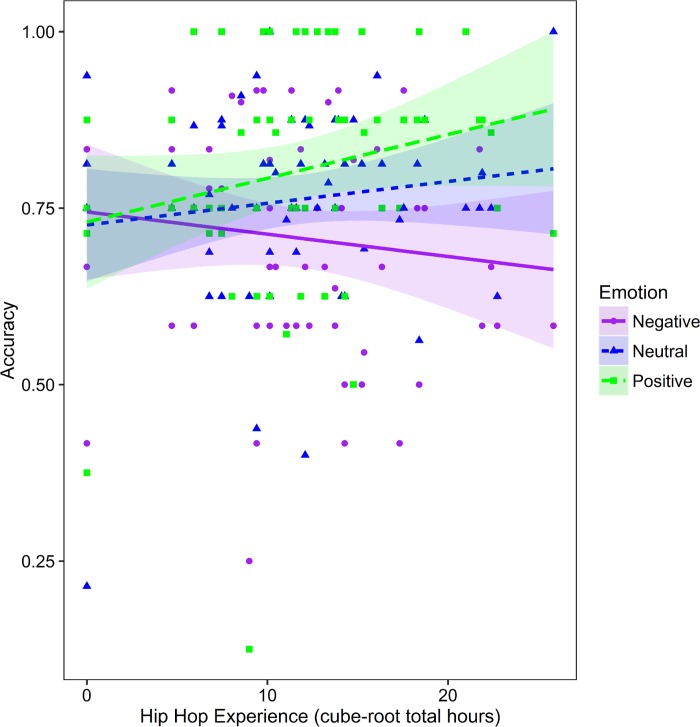
Relation between Reading the Mind in the Eyes task performance and hip hop dance experience. Performance on the Reading the Mind in the Eyes task for each emotion condition as a function of hip hop dance expertise. Shaded areas indicate 95% CI.

A reduced logistic mixed model using the ‘*glmer’* function with age, gender, RT, emotion (reference condition = negative), hip hop dance experience, and other dance style experience as fixed effects, random slope and intercept for participant (by emotion and RT) and random intercept for stimulus image was conducted. Due to limitations with regard to estimating the ANOVA table for a binomial dependent variable, the main effects and interactions were estimated using contrasts (emotion: negative = -1; neutral = 0, positive = 1) and then post-hoc comparisons were examined using the detailed linear mixed model (see Table E in S1 File). A significant main effect of age was observed (higher accuracy for older individuals) and for RT (higher accuracy on trials with longer RTs; see Table 6). A significant interaction was observed between hip hop experience and emotion condition. However, the effect was driven by a marginal relation between hip hop dance and accuracy for the positive emotion trials, *p* = 0.133, such that accuracy increased with greater hip hop experience in contrast to the reference condition, negative emotion trials (Bonferroni-corrected for three comparisons; neutral versus negative emotion *p* = 0.427; see Fig 6).

**Table 6:** Main effects and interactions on the Reading the Mind in the Eyes (RME) task when predicting accuracy.

Effect	Estimate	SE	*z*-value	*p*-value
Intercept	1.386	0.151	9.189	<0.001***
Age	0.477	0.088	5.43	<0.001***
RT	-0.292	0.063	-4.625	<0.001***
Gender (Male)	-0.057	0.09	-0.634	0.526
Emotion	-0.258	0.137	-1.876	0.061
Hip Hop Hours	-0.023	0.092	-0.248	0.804
Other style Hours	-0.094	0.093	-1.012	0.311
Emotion: Hip Hop Hours	-0.13	0.061	-2.127	0.033*
Emotion: Other style Hours	0.002	0.062	0.04	0.968
Hip Hop Hours: Other style Hours	0.127	0.078	1.625	0.104
Emotion: Hip Hop Hours: Other style Hours	0.003	0.051	0.066	0.947

RT, reaction time; SE, standard error

*p<0.05

***p<0.001

## Discussion

Building off of previous gaming and kinesthetic expertise research, in the present study we examined whether hip hop dance experience was connected to sociocognitive abilities. Performance on a mental rotation task, and to a lesser extent on the RME, was connected with hip hop dance experience. For the mental rotation task, dancers with greater hip hop experience were faster at judging whether a visually presented hand was left or right at a greater angular disparity compared to a baseline orientation. For the RME task, there was a marginal effect: dancers with greater hip hop experience tended to be more accurate when identifying positive versus negative facial emotions. The specificity of the connection to mental rotation speed and more difficult hand-image orientations suggests this was not due to a more general factor (e.g., general intelligence). No connection with hip hop dance experience was observed for working memory capacity, nor the number of moves taken when solving a problem. We next consider how the results of the present study align with previous gaming and kinesthetic expertise research.

### Specificity of Links between Hip Hop Dance Experience and Sociocognitive Skills

#### Mental rotation

The specific connection between hip hop dance experience and embodied stimuli replicates and extends previous research. It has previously been observed that more experienced gymnasts are faster at making left-right judgments of body figures oriented at more extreme angles [33]. That a similar effect was observed in the present study suggests that hip hop dance is also connected to embodied mental rotation ability. This result does not align with a previous study with traditional dancers which did not find a connection between embodied mental rotation reaction time and dance experience [32]. This could be either due to differences in the specific type of rotation judgment with regard to rotated body images or the style of dance. With regard to the former, Steggmann and colleagues (2011) found that although there was a connection between gymnastic experience and speed of mental rotation judgments of body images, it was specific to when participants were asked to judge whether the figure was raising their right or left hand, similar to what was observed in the present study. No such connection was previously observed when participants made same-different judgments of identical stimuli. The present findings may have also deviated from previous research with classical dancers due to differences in dance styles. We did not observe a link between experience in other dance styles and embodied mental rotation speed, similar to the lack of a connection with traditional dance experience observed in Jola and Mast (2005). This suggests that unlike hip hop, experience in more traditional dance styles may not be reflected in embodied mental rotation judgments.

That hip hop dance experience predicted embodied mental rotation speed only at the most extreme orientation suggests that this connection may be strongest with more difficult body-specific rotations. As indicated by the main effect of condition, performance was greater for hand versus block stimuli which may have led to a ceiling effect for the less extreme orientation trials for the hand condition. As such, it is possible that the connection between hip hop dance, and potentially other dance styles, experience would have been stronger if more extreme orientations (e.g., > 150°) or more difficult judgments were required. By including more challenging judgments, future research can examine the extent to which dance experience is linked with embodied mental rotation performance.

The specificity of the link to rotated images of hands suggests that hip hop dance experience has limited connections with general mental rotation ability. When examining connections between cognitive processes and experience, transfer effects are typically categorized depending on whether they extend to skills that are immediately involved in the experience (near transfer) or to skills that are more peripherally related or general (far transfer; [84]). That kinesthetic experience in previous research, and the present study, has not been observed to enhance mental rotation judgments of artificial objects is in line with near rather than far transfer. This aligns with the view that individuals with kinesthetic experience may be better at mentally simulating different perspectives of human figures compared to artificial objects [33]. This raises the question as to whether a similar pattern may be observed between video game experience and mentally rotating block stimuli. It may be that advantages of video game experience depend on the type of stimuli presented within the game: experience with games that contain artificial figures (e.g., puzzle games) or human-like figures (e.g., first-person shooters) may be specifically linked with performance when mentally rotating block versus embodied stimuli, respectively. Future research that examines whether and which categories video game experience are linked with advantages when making judgments about body-specific figures could provide evidence as to whether gaming experience yields near and far transfer effects with regard to mental rotation ability.

#### Reading the mind in the eyes performance

We observed a marginal effect such that dancers with greater hip hop experience were better at identifying certain types of emotion states displayed via facial expressions. This trend was specific to a greater difference in accuracy between positive and negative emotions, identifying positive emotions more accurately than negative ones. This partially aligns with previous arts and humanities research, specifically that students with greater acting experience were more accurate overall on the RME task [4]. Although the effect was weak, this may suggest that related tasks and sociocognitive abilities could be more strongly connected to hip hop dance. For example, it may be that accumulated dance experience is connected to empathy, which has been found to be correlated to RME performance [48] and arts-related experience [49]. Furthermore, it is possible that self-oriented emotion processes may be more strongly linked to dance experience compared to recognition of emotion states in others. For example, emotion regulation, where individuals modulate their emotion reactions to stimuli, is likely utilized by multiple dance styles including hip hop and modern and could be connected to accumulated dance experience.

#### Working memory

The pattern of connections observed in the present study between hip hop dance experience and sociocognitive skills align with and deviate from previous research in several ways. First, the lack of a connection between hip hop dance experience and working memory capacity is in line with previous research that has found no connection with sports expertise [24,25]. This is in contrast to evidence that working memory capacity is connected to experience in different types of traditional and video games (2,18). Similar to previous kinesthetic expertise research, as well as the present study, spatial working memory tasks have been included in gaming research. Although it is possible that differences in the respective spatial working memory tasks could account for different results observed in kinesthetic and video game expertise research, given the strength of correlations between different working memory tasks and the general construct of working memory capacity [14,85], this seems unlikely. Alternatively, it may be that when practicing or teaching dance routines hip hop dancers in the present study relied more strongly upon a type of working memory that was not captured by the spatial operation span task. We selected this task in an attempt to capture dancers’ ability to navigate a dance floor with a particular spatial sequence and timing. However, there is evidence that other processes, in addition to visuospatial working memory, may be involved when retaining visuomotor information over short periods of time [86,87]. It is possible that, in future research, performance on a working memory task that more directly taps the retention of visuomotor information would be connected to dance experience.

#### Problem solving

Although we observed an effect of task difficulty, there was no significant connection between problem solving performance and dance experience. In previous research, experienced chess players were found to be more likely to find an optimal solution on the Tower of London task [38]. The lack of a significant connection to dance experience may reflect that dancers have experience with dance routines that vary in the level of planned structure. For example, hip hop dance includes multiple different types of performances, some of which, such as break dancing, are more spontaneous and in response to music, and others that are more structured, pre-choreographed routines. Similarly, dance routines for other styles, such as ballet and jazz, can also vary in the level of structure and improvisation. Future research should examine whether problem solving ability is specifically related to experience with improvisational versus structured dance routines.

### Causal Directions and Future Research

In the present study we found evidence that hip hop dance experience was associated with better performance when mentally rotating images of hands. Given that previous research has provided evidence that training individuals on video games and acting can lead to improvements in visuospatial and theory of mind ability, respectively [3,49], it is possible that hip hop dance experience could lead to changes in mental rotation skills. However, this assumes that the causal direction for the associations observed in the present study are such that hip hop experience leads to changes in mental rotation ability. The reverse direction is also plausible: individuals who have greater mental rotation ability for human figures may self-select to gain experience with hip hop dance. Although previous research suggests that hip hop dance programs can lead to changes in self-perception [53], it remains to be seen if this experience is also reflected in changes to sociocognitive skills, and potentially academic achievement. Future research using training paradigms where participants are given experience with hip hop dance can help elucidate the causal direction of the link between hip hop dance experience and select sociocognitive skills.

Given the exploratory nature of the present study, future research should specifically target expert hip hop dancers from different geographical locations to examine the extent to which this experience is related to mental rotation ability. In the present study, we recruited a range of dancers who varied in hip hop dance experience. However, we were limited in the number of expert hip hop dancers we were able to recruit within the geographical area in which the study took place. As such it remains an open question as to whether the connections between embodied mental rotation and hip hop dance hours observed in the present study will extend to more expert compared to beginner and mid-level dancers. To recruit a sample of highly experienced hip hop dancers we recommend that future studies target hip hop dance events (e.g., expositions, conferences) where it is highly likely for a large sample of experienced dancers to be available for recruitment (see [88] for a parallel example in video game research). Furthermore, given the variability in the styles of hip hop dance [58], it also remains to be seen whether connections between dance experience and mental rotation ability varies across geographic regions. Even across different cities within the United States of America there are large variations in which hip hop dance styles are popular (personal communication). Given that the present study was conducted with dancers in the Pacific Northwest region of the United States of America, it remains to be seen whether similar, or different, connections between hip hop dance experience and sociocognitive abilities are observed with dancers from other regions.

Future research should also more explicitly compare the kinesthetic properties of hip hop dance to other styles. There is limited research documenting the kinesthetic characteristics of dance moves frequently used in hip hop dance and how they differ from other dance styles. Evidence that hip hop and other dance style experience differentially correlated with mental rotation skills in the present study raises questions regarding the source of these differences. One possible source could be the kinesthetic differences in the frequency and types of movements used across different dance styles. In contrast, it could be due to the motivations and environments in which different dance styles take place. Research that specifically examines how different dance styles vary across these aspects can inform how different connections to sociocognitive skills may emerge.

With high school students in the United States continuing to lag behind their international peers in math and science [89] there is continued interest in how science, technology, engineering, and math (STEM) skills can be improved. That individual differences in cognitive processes related to STEM skill can be enhanced via experience in gaming and kinesthetic activities [15,84] suggests that, in additional to formal education, informal activities may contribute to improvements in STEM ability. Although programs in schools and extracurricular activities that aim to provide creative outlets for students to enhance STEM skills tend to focus on robotics, game programming, and other technical activities, the results of the present study suggest fine arts activities could also be utilized. Hip hop dance in particular seems well suited to engage students who many not be interested in participating in technical activities or other arts and humanities programs. By being relatively popular with lower socioeconomic status and minority groups, hip hop dance programs could attract students from these groups. Although hip hop dance would likely not be the environment in which academic skills related to STEM are learned, it is possible that this type of experience could refine underlying sociocognitive skills. Since the sample demographics of the present study were mainly young adults, future research should examine whether a similar set of connections are present with K-12 student populations from under-represented groups.

## Conclusion

In the present study, we explored whether individual differences in performance on tasks tapping different sociocognitive abilities were connected to hip hop dance experience. Motivated by previous video gaming and kinesthetic expertise research, we presented dancers with a working memory, mental rotation, problem solving, and emotion recognition task. Dancers with greater hip hop experience had significantly better performance when mentally rotating difficult embodied images and were marginally better at identifying positive versus negative emotion states displayed by faces. We suggest that these results indicate that hip hop dance experience is connected to specific sociocognitive skills. We discuss ways in which future research can build upon the present study, such as examining the causal direction of the observed links between hip hop dance experience and sociocognitive skills, whether these connections are observed with dancers in different geographic regions and age groups, and if hip hop dance training could be used to supplement STEM education.

## Supporting Information

S1 File Supporting Information. Fixed and random effects when predicting working memory task performance (Table A). Fixed effects when predicting mental rotation task performance. (Table B). Random effects when predicting mental rotation task performance (Table C). Fixed and random effects when predicting Tower of London task performance (Table D). Fixed and random effects when predicting Reading the Mind in the Eyes (RME) task performance (Table E).
(DOCX)
