# Supplementary material for: Hip Hop Dance Experience Linked to Sociocognitive Ability
Source: PLoS One. 2017 Feb 1;12(2):e0169947. doi: 10.1371/journal.pone.0169947 (PMC5287481; doi:10.1371/journal.pone.0169947)
Supplement: S1 File — Fixed and random effects when predicting working memory task performance (Table A). Fixed effects when predicting mental rotation task performance. (Table B). Random effects when predicting mental rotation task performance (Table C). Fixed and random effects when predicting Tower of London task performance (Table D). Fixed and random effects when predicting Reading the Mind in the Eyes (RME) task performance (Table E). (DOCX) [file pone.0169947.s001.docx]

**Supporting Information**

**Tower of London Task Stimuli**

The ID numbering system of Berg and Byrd (2002) for the start and goal boards used during test trials was as follows: four-moves (move = 42, goal = 55; move = 45, goal = 51), five-moves (move = 23, goal = 41; move = 45, goal = 25), seven-moves (move = 55, goal = 21; move = 63, goal = 35).

**Working Memory**

A maximal linear mixed model with age, gender (female used as reference), set length (3-item set length used as reference), hip hop dance experience, and other dance style experience as fixed effects, random slope and intercept for participant (by set length) revealed a high correlation (1.00) between the random intercept and slope, suggesting over-parameterization. Simplifying the model by removing the random slope revealed, however, that the maximal model was a better fit (maximal: log-likelihood = -448.82, AIC = 933.64, BIC = 1002.29; reduced: log-likelihood = -465.99, AIC = 955.97, BIC = 1001.74; *Χ^2^*[6] = 34.33, *p* < 0.001), and combined with a moderate effect size, *Ω^2^_0_* = 0.374, suggested the maximal model should be retained (see Table A).

**Table A.** **Fixed and random effects when predicting working memory task performance. Degrees of freedom and p-values approximated using Satterthwaite method.**

| **Fixed Effects** | |  | **Working Memory: Dependent Variable = Block Score** | | | | |
| --- | --- | --- | --- | --- | --- | --- | --- |
|  |  |  | ***Estimate*** | ***SE*** | ***Approx. DF*** | ***t-value*** | ***Approx. p*** |
|  | Intercept |  | -0.235 | 0.084 | 276.618 | -2.795 | 0.006** |
|  | Age |  | 0.019 | 0.065 | 121.390 | 0.295 | 0.768 |
|  | Gender (Male) |  | 0.083 | 0.068 | 114.562 | 1.226 | 0.223 |
|  | Hip Hop Hours |  | -0.001 | 0.089 | 291.438 | -0.007 | 0.995 |
|  | Set length (4-item) |  | 0.354 | 0.131 | 245.342 | 2.702 | 0.007** |
|  | Set length (5-item) |  | 0.359 | 0.166 | 70.217 | 2.167 | 0.034* |
|  | Other style Hours |  | -0.061 | 0.092 | 288.746 | -0.668 | 0.505 |
|  | Hip Hop Hours : Set length (4-item) |  | -0.022 | 0.136 | 250.815 | -0.164 | 0.87 |
|  | Hip Hop Hours : Set length (5-item) |  | -0.090 | 0.170 | 68.942 | -0.530 | 0.598 |
|  | Hip Hop Hours : Other style Hours |  | -0.036 | 0.072 | 298.976 | -0.497 | 0.619 |
|  | Set length (4-item) : Other style Hours |  | -0.012 | 0.141 | 258.166 | -0.085 | 0.933 |
|  | Set length (5-item) : Other style Hours |  | -0.149 | 0.182 | 76.965 | -0.816 | 0.417 |
|  | Hip Hop Hours : Set length (4-item) : Other style Hours |  | 0.063 | 0.110 | 260.037 | 0.571 | 0.568 |
|  | Hip Hop Hours : Set length (5-item) : Other style Hours |  | 0.044 | 0.140 | 70.809 | 0.311 | 0.756 |
|  |  |  |  |  |  |  |  |
|  |  |  |  | **Random Effects** |  |  |  |
|  |  |  |  |  |  | ***Estimate*** | ***SD*** |
|  |  |  |  | Participant |  |  |  |
|  |  |  |  |  | Intercept | 0.135 | 0.367 |
|  |  |  |  |  | Set length | 0.111 | 0.333 |
|  |  |  |  | Residual |  | 0.706 | 0.840 |

SE, standard error; DF, degrees of freedom

^*^*p*<0.05

^**^*p*<0.001

**Mental Rotation**

A maximal linear mixed model with age, gender (female used as reference), orientation (0°used as reference), stimulus type (dummy coded, block condition as reference), hip hop dance experience, accuracy, and other dance style experience as fixed effects, random slope and intercept for participant (by orientation, stimulus type, and accuracy) and random intercept for stimulus image revealed a large effect size, *Ω^2^_0_* = 0.795 (log-likelihood = -2092.16, AIC = 4246.31, BIC = 4430.53), suggesting the maximal model should be retained.

The significant interaction between hip hop dance experience, condition, and orientation was further analyzed using the full model that analyzed the link between reaction time and hip hop experience for each condition and orientation level with regard to the reference (reference for orientation = 0°; reference for condition = Block; see Tables B and C). Although there were other significant effects in the full model, they did not warrant further inspection as indicated by non-significant effects in the mixed-factors ANOVA (see main text).

**Table B.** **Fixed effects when predicting mental rotation task performance. Degrees of freedom and p-values approximated using Satterthwaite method.**

| **Fixed Effects** | | **Mental Rotation: Dependent Variable = Reaction Time** | | | | | |
| --- | --- | --- | --- | --- | --- | --- | --- |
|  |  |  | ***Estimate*** | ***SE*** | ***DF*** | ***t-value*** | ***p-value*** |
|  | Intercept |  | 0.535 | 0.097 | 71.321 | 5.527 | <0.001*** |
|  | Age |  | 0.059 | 0.043 | 62.449 | 1.377 | 0.173 |
|  | Gender (Male) |  | -0.293 | 0.105 | 61.151 | -2.792 | 0.007** |
|  | Accuracy |  | 0.030 | 0.012 | 50.797 | 2.505 | 0.016* |
|  | Orientation (50) |  | 0.263 | 0.115 | 40.391 | 2.292 | 0.027* |
|  | Orientation (100) |  | 0.505 | 0.111 | 42.466 | 4.536 | <0.001*** |
|  | Orientation (150) |  | 0.495 | 0.123 | 41.495 | 4.040 | <0.001*** |
|  | Condition (Hand) |  | -1.318 | 0.132 | 65.764 | -9.948 | <0.001*** |
|  | Hip Hop Hours |  | -0.071 | 0.059 | 76.012 | -1.191 | 0.237 |
|  | Other style Hours |  | -0.049 | 0.061 | 74.257 | -0.809 | 0.421 |
|  | Orientation (50) : Condition (Hand) |  | -0.364 | 0.162 | 40.341 | -2.242 | 0.03* |
|  | Orientation (100) : Condition (Hand) |  | -0.398 | 0.156 | 41.083 | -2.553 | 0.014* |
|  | Orientation (150) : Condition (Hand) |  | -0.128 | 0.171 | 39.699 | -0.747 | 0.459 |
|  | Orientation (50) : Hip Hop Hours |  | 0.075 | 0.039 | 2602.536 | 1.935 | 0.053 |
|  | Orientation (100) : Hip Hop Hours |  | -0.007 | 0.041 | 502.260 | -0.169 | 0.866 |
|  | Orientation (150) : Hip Hop Hours |  | 0.107 | 0.044 | 209.239 | 2.450 | 0.015* |
|  | Condition (Hand) : Hip Hop Hours |  | 0.095 | 0.078 | 89.247 | 1.221 | 0.225 |
|  | Orientation (50) : Other style Hours |  | 0.078 | 0.041 | 2563.546 | 1.906 | 0.057 |
|  | Orientation (100) : Other style Hours |  | 0.057 | 0.044 | 505.487 | 1.299 | 0.195 |
|  | Orientation (150) : Other style Hours |  | 0.048 | 0.046 | 206.621 | 1.032 | 0.303 |
|  | Condition (Hand) : Other style Hours |  | -0.035 | 0.081 | 89.836 | -0.432 | 0.667 |
|  | Hip Hop Hours : Other style Hours |  | -0.038 | 0.053 | 76.522 | -0.724 | 0.471 |
|  | Orientation (50) : Condition (Hand) : Hip Hop Hours |  | -0.069 | 0.054 | 2595.496 | -1.271 | 0.204 |
|  | Orientation (100) : Condition (Hand) : Hip Hop Hours |  | -0.014 | 0.055 | 2621.358 | -0.257 | 0.797 |
|  | Orientation (150) : Condition (Hand) : Hip Hop Hours |  | -0.158 | 0.055 | 2623.253 | -2.869 | 0.004** |
|  | Orientation (50) : Condition (Hand) : Other style Hours |  | -0.060 | 0.057 | 2597.158 | -1.056 | 0.291 |
|  | Orientation (100) : Condition (Hand) : Other style Hours |  | -0.011 | 0.057 | 2624.160 | -0.196 | 0.844 |
|  | Orientation (150) : Condition (Hand) : Other style Hours |  | -0.029 | 0.057 | 2636.036 | -0.510 | 0.61 |
|  | Orientation (50) : Hip Hop Hours : Other style Hours |  | 0.074 | 0.034 | 1681.800 | 2.149 | 0.032* |
|  | Orientation (100) : Hip Hop Hours : Other style Hours |  | 0.019 | 0.037 | 477.737 | 0.505 | 0.614 |
|  | Orientation (150) : Hip Hop Hours : Other style Hours |  | 0.025 | 0.038 | 201.517 | 0.647 | 0.518 |
|  | Condition (Hand) : Hip Hop Hours : Other style Hours |  | -0.060 | 0.066 | 92.355 | -0.909 | 0.366 |
|  | Orientation (50) : Condition (Hand) : Hip Hop Hours : Other style Hours |  | -0.080 | 0.046 | 2384.373 | -1.725 | 0.085 |
|  | Orientation (100) : Condition (Hand) : Hip Hop Hours : Other style Hours |  | -0.003 | 0.047 | 2550.661 | -0.063 | 0.95 |
|  | Orientation (150) : Condition (Hand) : Hip Hop Hours : Other style Hours |  | -0.025 | 0.047 | 2569.412 | -0.533 | 0.594 |
|  |  |  |  |  |  |  |  |

SE, standard error; DF, degrees of freedom

^*^*p*<0.05

^**^*p*<0.001

^***^*p*<0.001

**Table C.** **Random effects when predicting mental rotation task performance.**

| **Random Effects** |  |  |  |
| --- | --- | --- | --- |
|  |  | ***Estimate*** | ***SD*** |
| Participant |  |  |  |
|  | Intercept | 0.088 | 0.296 |
|  | Accuracy | 0.002 | 0.041 |
|  | Orientation | 0.057 | 0.239 |
|  | Condition | 0.003 | 0.054 |
| Stimulus Image |  |  |  |
|  | Intercept | 0.029 | 0.171 |
| Residual |  | 0.218 | 0.466 |

SD, standard deviation

**Tower of London**

A maximal linear mixed model with age, gender (female used as reference), minimum number of moves (4 moves used as reference), first move RT, hip hop dance experience, and other dance style experience as fixed effects, random slope and intercept for participant (by minimum number of moves, first move RT) and random intercept for stimulus type revealed a strong correlation (-1.00) between the random intercept and slope, suggesting over-parameterization (maximal: log-likelihood = -421.06, AIC = 888.12, BIC = 974.72). Simplifying the model by removing the random slope revealed a similar fit (reduced: log-likelihood = -421.78, AIC = 879.56, BIC = 947.33), and combined with a moderate effect size, *Ω^2^_0_* = 0.217, suggested the reduced model should be retained (see Table D).

**Table D.** **Fixed and random effects when predicting Tower of London task performance. Degrees of freedom and p-values approximated using Satterthwaite method.**

| **Fixed Effects** | | **Tower of London: Dependent Variable = Number of Extra Moves** | | | | | |
| --- | --- | --- | --- | --- | --- | --- | --- |
|  |  |  | ***Estimate*** | ***SE*** | ***Approx. DF*** | ***t-value*** | ***Approx. p*** |
|  | Intercept |  | -0.448 | 0.093 | 7.658 | -4.822 | 0.001** |
|  | Age |  | -0.088 | 0.059 | 68.961 | -1.488 | 0.141 |
|  | Gender (Male) |  | -0.048 | 0.064 | 53.379 | -0.749 | 0.457 |
|  | First RT |  | -0.112 | 0.057 | 265.172 | -1.982 | 0.048* |
|  | Hip Hop Hours |  | 0.061 | 0.101 | 228.934 | 0.607 | 0.544 |
|  | Minimum Moves (5) |  | 0.808 | 0.135 | 8.678 | 6.007 | <0.001*** |
|  | Minimum Moves (7) |  | 0.555 | 0.134 | 8.521 | 4.147 | 0.003** |
|  | Other style Hours |  | 0.089 | 0.095 | 228.925 | 0.932 | 0.352 |
|  | Hip Hop Hours : Minimum Moves (5) |  | 0.012 | 0.132 | 258.265 | 0.091 | 0.928 |
|  | Hip Hop Hours : Minimum Moves (7) |  | -0.029 | 0.134 | 258.734 | -0.213 | 0.831 |
|  | Hip Hop Hours : Other style Hours |  | 0.080 | 0.089 | 265.230 | 0.895 | 0.372 |
|  | Minimum Moves (5) : Other style Hours |  | -0.197 | 0.131 | 256.012 | -1.507 | 0.133 |
|  | Minimum Moves (7) : Other style Hours |  | 0.106 | 0.135 | 266.167 | 0.783 | 0.434 |
|  | Hip Hop Hours : Minimum Moves (5) : Other style Hours |  | -0.198 | 0.117 | 260.241 | -1.688 | 0.093 |
|  | Hip Hop Hours : Minimum Moves (7) : Other style Hours |  | -0.182 | 0.138 | 291.783 | -1.317 | 0.189 |
|  |  |  |  |  |  |  |  |
|  |  |  |  | **Random Effects** |  |  |  |
|  |  |  |  |  |  | ***Estimate*** | ***SD*** |
|  |  |  |  | Participant |  |  |  |
|  |  |  |  |  | Intercept | 0.023 | 0.153 |
|  |  |  |  | Trial |  |  |  |
|  |  |  |  |  | Intercept | 0.000 | 0.021 |
|  |  |  |  | Residual |  | 0.802 | 0.895 |

RT, reaction time; SE, standard error; DF, degrees of freedom

^*^*p*<0.05

^**^*p*<0.001

^***^*p*<0.001

**Reading the Mind in the Eyes Task**

A maximal logistic mixed model using the ‘*glmer’* function with age, gender (female used as reference), RT, emotion (negative used as reference), hip hop dance experience, and other dance style experience as fixed effects, random slope and intercept for participant (by emotion and RT) and random intercept for stimulus image failed to converge (maximal: log-likelihood = -1088.23, AIC = 2220.47, BIC = 2345.22). Simplifying the model by removing the random slope revealed a similar fit (reduced: log-likelihood = -1095.21, AIC = 2216.43, BIC = 2290.14), suggesting the reduced model should be retained (see Table E).

**Table E.** **Fixed and random effects when predicting Reading the Mind in the Eyes (RME) task performance. Degrees of freedom and p-values approximated using logistic mixed model.**

| **Fixed Effects** | |  | **RME: Dependent Variable = Accuracy** | | | |
| --- | --- | --- | --- | --- | --- | --- |
|  |  |  | ***Estimate*** | ***SE*** | ***t-value*** | ***Approx. p*** |
|  | Intercept |  | 1.056 | 0.241 | 4.389 | <0.001*** |
|  | Age |  | 0.478 | 0.088 | 5.435 | <0.001*** |
|  | RT |  | -0.293 | 0.063 | -4.641 | <0.001*** |
|  | Gender (Male) |  | -0.057 | 0.090 | -0.632 | 0.527 |
|  | Emotion (Neutral) |  | 0.401 | 0.310 | 1.296 | 0.195 |
|  | Emotion (Positive) |  | 0.683 | 0.378 | 1.808 | 0.071 |
|  | Hip Hop Hours |  | -0.186 | 0.119 | -1.565 | 0.118 |
|  | Other style Hours |  | -0.074 | 0.121 | -0.613 | 0.54 |
|  | Emotion (Neutral) : Hip Hop Hours |  | 0.193 | 0.132 | 1.467 | 0.142 |
|  | Emotion (Positive) : Hip Hop Hours |  | 0.344 | 0.171 | 2.010 | 0.044* |
|  | Emotion (Neutral) : Other style Hours |  | -0.047 | 0.136 | -0.344 | 0.731 |
|  | Emotion (Positive) : Other style Hours |  | 0.007 | 0.173 | 0.040 | 0.968 |
|  | Hip Hop Hours : Other style Hours |  | 0.122 | 0.102 | 1.197 | 0.231 |
|  | Emotion (Neutral) : Hip Hop Hours : Other style Hours |  | 0.018 | 0.110 | 0.159 | 0.873 |
|  | Emotion (Positive) : Hip Hop Hours : Other style Hours |  | -0.012 | 0.143 | -0.082 | 0.935 |
|  |  |  |  |  |  |  |
|  |  |  | **Random Effects** | |  |  |
|  |  |  |  |  | ***Estimate*** | ***SD*** |
|  |  |  | Participant |  |  |  |
|  |  |  |  | Intercept | 0.174 | 0.417 |
|  |  |  | Trial |  |  |  |
|  |  |  |  | Intercept | 0.539 | 0.734 |

RT, reaction time; SE, standard error; DF, degrees of freedom

^*^*p*<0.05

^***^*p*<0.001
